# Effect of vitamin D_3_ on antiphospholipid antibodies in hospitalized patients with moderate to severe COVID-19

**DOI:** 10.1016/j.clinsp.2024.100474

**Published:** 2024-08-28

**Authors:** Lucas P. Sales, Lucas V.B. Souza, Alan L. Fernandes, Igor H. Murai, Mayara D. Santos, Margarete B.G. Vendramini, Ricardo M. Oliveira, Camille P. Figueiredo, Valéria F. Caparbo, Bruno Gualano, Rosa M.R. Pereira, in memoriam

**Affiliations:** a Rheumatology Division, Hospital das Clínicas, Faculdade de Medicina, Universidade de São Paulo (HCFMUSP), São Paulo, SP, Brazil; b RDO Diagnósticos Médicos, São Paulo, SP, Brazil

**Keywords:** COVID-19, SARS-CoV-2 Infection, Vitamin D, Antiphospholipid Antibodies Syndrome, Antiphospholipid Antibodies

## Abstract

**Objective:**

To investigate the effect of a single oral dose of 200,000 IU of vitamin D_3_ on antiphospholipid antibodies in hospitalized patients with moderate to severe COVID-19.

**Methods:**

This is a post-hoc, exploratory analysis from a double-blind, placebo-controlled, randomized clinical trial performed in two centers in Sao Paulo, Brazil. Hospitalized patients with COVID-19 were randomly assigned to receive either vitamin D_3_ (*n* = 97) or placebo (*n* = 97). In this post-hoc analysis, the endpoints were titers and frequency of anti–β2-Glycoprotein-I (aβ2-GP) and Anticardiolipin (aCL) antibodies [Immunoglobulin G, M and A (IgG, IgM and IgA)].

**Results:**

Overall mean (SD) age was 55.3 (13.9) years, Body Mass Index (BMI) was 32.2 (7.1 kg/m^2^), and 106 participants (54.6 %) were male. There was a significant group by time interaction (*p* = 0.046) for frequency of aCL IgG, with increased values from baseline to discharge in the placebo group [n (%), from 13 (13.4) to 25 (25.8)] compared to the vitamin D_3_ [from 25 (25.8) to 29 (29.9)]. However, the frequency of aCL IgG did not change between the groups on discharge. No significant differences between vitamin D3 and placebo groups were found for any other autoantibodies.

**Conclusion:**

These findings do not support the use of a single oral dose of 200,000 IU of vitamin D_3_ to modulate autoantibodies in hospitalized patients with moderate to severe COVID-19.

## Highlights

• The impact of vitamin D_3_ supplementation on autoimmunity remains a subject of debate.
• A single dose of 200,000 IU of vitamin D_3_ was not able to modulate autoantibodies in COVID-19 patients.
• aPL antibody positivity was not associated with thrombotic events despite vitamin D3 supplementation.
• aPL antibodies associated with the virus seem to be transient in critical patients.

## Introduction

Since the beginning of the Coronavirus Disease 2019 (COVID-19) pandemic, caused by Severe Acute Respiratory Syndrome Coronavirus 2 (SARS-CoV-2), a possible relationship between the infection and autoimmune reactions has been hypothesized.^1^ In addition to the most common clinical manifestations (i.e., fever, cough, and dyspnea), the presence of myalgia, joint pain (arthralgia), and thrombotic events was recurrent in hospitalized patients.^2^, ^3^, ^4^ One of the explanations is the fact that SARS-CoV-2 infection affects regulatory T-cell levels, aggravating inflammatory processes that may lead to autoimmunity.^5^

In this context, Zhang et al.^6^ described three cases of thrombosis associated with both Antiphospholipid (aPL) antibodies, Anticardiolipin (aCL), and anti–β2-Glycoprotein-I (aβ2-GP). These antibodies target phospholipid-binding proteins and phospholipids in cell membranes, leading to a hypercoagulable state through interference with the coagulation cascade, activation of endothelial cells and platelets, and inhibition of natural anticoagulant pathways.^7^

Pascolini et al.^8^ showed that the frequency of autoantibody positivity in the COVID-19 group was significantly higher than in the control group (45 % vs. 12 %; *p* = 0.03). The subgroup of patients testing positive for autoantibodies exhibited higher lactate levels and a poorer prognosis compared to the subgroup testing negative. The mortality rate due to COVID-19 complications was significantly higher in the autoantibody-positive subgroup compared to the negative subgroup (40 % vs. 5.5 %; *p* = 0.03).

The role of vitamin D_3_ supplementation in autoimmunity has been extensively investigated. In addition to influencing viral neutralization, recruitment of immune system cells and dendritic cell maturation,^9^^,^^10^ vitamin D_3_ acts on lymphocytes T and B proliferation, immune cells that participate in antibody production.^11^^,^^12^ For instance, Najafipoor et al.^13^ demonstrated that patients who received 50,000 IU of vitamin D_3_ per week for 6 months, in addition to interferon therapy, presented lower increases in IgG levels against Epstein-Barr virus 1 and viral capsid antigen compared to the control group, who only received disease-modifying interferon therapy alone. These findings align with previous evidence on the importance of adequate serum levels of vitamin D to reduce disease activity and remission, and to enhance responsiveness to rheumatoid arthritis treatment.^14^

Altogether, these findings suggest that vitamin D deficiency could act as an environmental trigger contributing to B-cell hyperactivation, which could culminate in increased autoantibody production.^10^^,^^15^, ^16^, ^17^ Although numerous clinical trials worldwide have investigated the impact of vitamin D_3_ on COVID-19 outcomes,^17^, ^18^, ^19^, ^20^, ^21^ the role of vitamin D_3_ on aPL antibodies remains poorly understood. To fill this gap, the present exploratory study aimed to assess the effect of a single oral dose of 200,000 IU of vitamin D_3_ on aPL antibodies in hospitalized patients with moderate to severe COVID-19.

## Methods

### Study design

This is a *post-hoc*, exploratory analysis from a double-blind, placebo-controlled, randomized clinical trial performed in two centers in Sao Paulo, Brazil, and registered in ClinicalTrials.gov, NCT04449718. This study was conducted in accordance with the Declaration of Helsinki and approved by the ethics committee of both centers: Hospital das Clinicas da Faculdade de Medicina da Universidade de São Paulo and Hospital de Campo do Ibirapuera (Ethics Committee Approval Number 30959620.4.1001.0068). Before being admitted to the study, all patients provided written informed consent. This manuscript was reported according to the CONSORT guidelines. Additional information about the trial concept and design, patient recruitment, supplementation protocol and blindness, procedures, and endpoints has been previously published.^22^

### Participants

Patients were recruited from both hospitals from June 2, 2020 to August 27, 2020. The end of follow-up was on October 7, 2020. Inclusion criteria were age ≥ 18 years; confirmed diagnosis of COVID-19 by polymerase chain reaction testing for SARS-CoV-2 from nasopharyngeal swabs, or computed tomography scan findings consistent with those found in COVID-19 (bilateral multifocal ground-glass opacities ≥ 50 %); and flu syndrome with institutional criteria for hospitalization on hospital admission (respiratory rate > 24 breaths/minute, oxygen saturation < 93 % on room air, or the presence of risk factors for complications (e.g., obesity, diabetes, systemic arterial hypertension, neoplasms, immunosuppression, heart disease, pulmonary tuberculosis), followed by COVID-19 confirmation. Patients who met these criteria were considered to have moderate to severe COVID-19 according to the criteria from the NIH-COVID-19 Treatment Guidelines.^23^

Patients were excluded if they were unable to read and sign the written informed consent form; were already under invasive mechanical ventilation; received prior vitamin D_3_ supplementation (> 1000 IU/d or weekly equivalent); had kidney failure requiring dialysis or creatinine > 2.0 mg/dL or hypercalcemia (calcium > 10.5 mg/dL); were pregnant or lactating women; were expecting to be discharged within 24 h. Absence of fever in the previous 72 h, no need for supplemental oxygen in the previous 48 h, and oxygen saturation greater than 93 % on room air without respiratory distress were used as criteria for hospital discharge.

### Randomization and masking

Patients were allocated in a 1:1 ratio into the vitamin D_3_ or placebo groups as previously described.^22^ Patients enrolled in the vitamin D_3_ group received a single oral dose of 200,000 IU of vitamin D_3_ diluted in vehicle (10 mL of a peanut oil solution) immediately after randomization, while those in the placebo group received only vehicle. Both solutions were prepared by the same unit (pharmacy of Hospital das Clínicas) and were identical in appearance, color, smell, and taste. This selected dose is within the recommended dose range for effectively promoting vitamin D sufficiency.^24^

### Procedures

Baseline demographic, self-reported anthropometric (weight and height), and clinical characteristics (coexisting chronic diseases, acute COVID-19 symptoms, patients’ medications throughout hospitalization, oxygen supplementation requirement, and imaging features) were collected upon hospital admission. Serum levels of 25-hydroxyvitamin D were assessed by chemiluminescent immunoassay (ARCHITECT 25-OH Vitamin D 5P02; Abbott Diagnostics). The frequency of thrombotic events during hospitalization was assessed through electronic medical records. The presence and titers of aCL antibodies (IgG, IgM and IgA) were analyzed through commercial fluoro immunoenzymatic assay (Thermo Scientific™/Phadia™ 250 Immunoassay Analyzers). The aβ2-GP antibodies (IgG, IgM and IgA) were detected by ELISA using a commercial kit (Quanta Lite®, Inova Diagnostics Ins., San Diego, CA, USA). Only patients who had blood samples collected on the day of randomization and on hospital discharge were included in this study. Patients who died throughout follow-up were not included due to the absence of blood samples.

### Outcome measures

Herein, it was reported on the following *post hoc* exploratory outcomes: presence and titers of aPL antibodies: aCL (IgG, IgM and IgA) and aβ2-GP (IgG, IgM and IgA).

### Statistical analysis

The sample size was chosen based on feasibility and resources, as previously described.^22^ Independent *t*-test and Mann-Whitney *U* test were used for continuous variables. Proportions were analyzed by chi-square or Fisher's exact test. Generalized Estimating Equations (GEE) for repeated measures were used for testing possible differences in serum presence and titers of autoantibodies assuming group and time as fixed factors, with marginal and binomial distributions, and a first-order autoregressive correlation matrix to test the main and interaction effects. Bonferroni's adjustment was performed in GEE analyses to maintain a family-wise two-sided significance threshold of 0.05, considering 6 pairwise comparisons for all outcomes. All analyses were performed by a per-protocol approach with no imputation for missing, data using IBM-SPSS software, version 20.0. The significance level was set at a two-sided p-value ≤ 0.05.

## Results

**Fig. 1:**
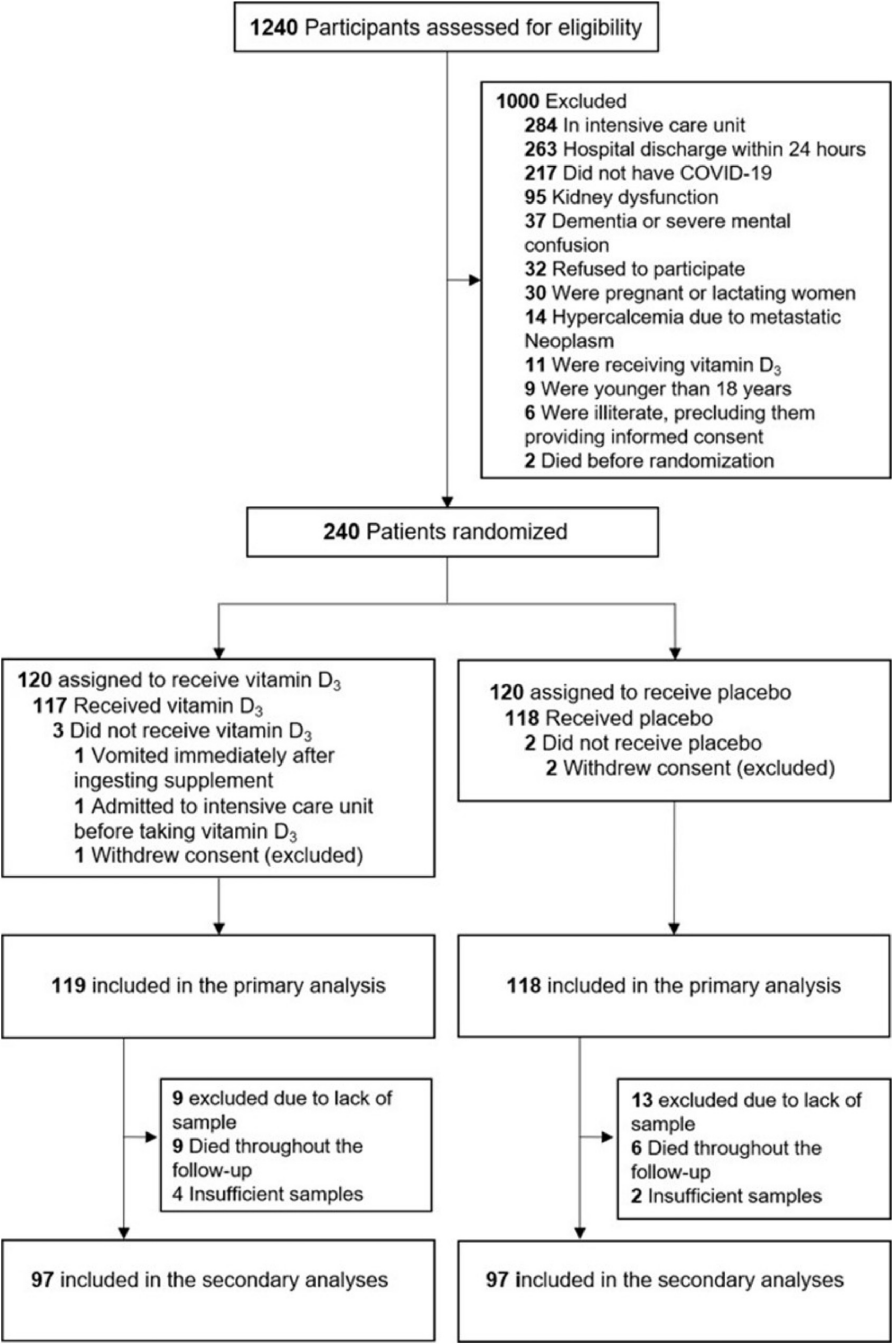
Trial CONSORT diagram.

A total of 1240 patients were screened for eligibility; however, only 240 were randomized during the acute-phase of SARS-CoV-2 infection, with 120 being assigned to each group. Three patients withdrew their informed consent. Of the 119 patients who were randomized to the vitamin D3 group, 13 (11 %) were excluded due to insufficient blood samples, and 9 (7 %) died throughout the follow-up. From 118 patients who were randomized to the placebo group, 15 (13 %) were excluded due to insufficient blood samples, and 6 (5 %) died throughout the follow-up (Fig. 1).

The mean (SD) age was 55.3 (13.9) years, Body Mass Index (BMI) was 32.2 (7.1) kg/m^2^, 106 (54.6 %) patients were male, 108 (55.7 %) were white, and 175 (90.2 %) required respiratory support at baseline. No significant differences between groups were found at baseline (Table 1).

**Table 1:** Baseline demographic and clinical characteristics.

Characteristic	Vitamin D3 group (*n* = 97)	Placebo group (*n* = 97)
Age, years	55.2 ± 13.8	55.4 ± 14.3
Sex, n (%)		
Male	57 (58.8)	49 (50.5)
Female	40 (41.2)	48 (49.5)
Race or ethnicity, n (%)		
White	49 (50.5)	59 (60.8)
Pardo^a^	31 (32.0)	28 (28.9)
Black	16 (16.5)	10 (10.3)
Asian	1 (1.0)	0 (0)
BMI, kg/m^2^^b^	32.3 ± 6.8	32.1 ± 7.6
Time for hospital length of stay, days	6.0 (4.0-8.0)	7.0 (6.0-10.0)
Concomitant medications, n (%)		
Anticoagulant^c^	90 (93.8)	83 (85.6)
Antibiotic^c^	81 (84.4)	84 (86.6)
Glucocorticoid^c^	65 (67.7)	62 (63.9)
Antihypertensive^c^	55 (57.3)	43 (44.3)
Proton pump inhibitorc	40 (41.7)	40 (41.2)
Antiemetic^c^	38 (39.6)	49 (50.5)
Analgesic^c^^,^^d^	37 (38.5)	45 (46.9)
Hypoglycemic^c^	23 (24)	20 (20.6)
Hypolipidemic^c^	14 (14.6)	14 (14.4)
Thyroid^c^	8 (8.3)	8 (8.2)
Antiviral^c^	4 (4.2)	3 (3.1)
Acute COVID-19 symptoms, n (%)		
Fever	70 (72.2)	68 (70.1)
Cough	83 (85.6)	81 (83.5)
Fatigue	80 (82.5)	84 (86.6)
Joint pain	40 (41.2)	33 (34)
Myalgia	60 (61.9)	60 (61.9)
Nasal congestion	33 (34)	33 (34)
Runny nose	34 (35.1)	36 (37.1)
Sore throat	35 (36.1)	23 (23.7)
Diarrhea	32 (33)	40 (41.2)
Coexisting diseases, n (%)		
Hypertension	53 (54.6)	47 (48.5)
Cardiovascular disease	14 (14.4)	12 (12.4)
Diabetes	39 (40.2)	28 (28.9)
Chronic obstructive pulmonary disease	5 (5.2)	5 (5.2)
Asthma	6 (6.2)	7 (7.2)
Obesity	56 (57.7)	56 (58.3)
Rheumatic disease	10 (10.3)	9 (9.3)
Chronic kidney disease	2 (2.1)	0 (0)
Respiratory support, n (%)		
No oxygen therapy	14 (14.4)	5 (5.2)
Oxygen therapy	70 (72.2)	81 (83.5)
Non-invasive ventilation	13 (13.4)	11 (11.3)
Computed tomography findings, n (%)^e^		
Ground-glass opacities < 50 %	38 (44.7)	31 (38.3)
Ground-glass opacities ≥ 50 %	47 (55.3)	50 (61.7)
Laboratory values^f^		
Platelet, 10^3^/µL	313 ± 114	299 ± 133
Mean platelet volume, Fl	10.5 ± 0.4	10.7 ± 0.9
ESR, mm/h	56.0 (24.0‒86.0)	61.0 (35.5‒88.0)
C-reactive protein, mg/L	56.9 (22.3‒92.7)	65.9 (28.8‒103.4)
D-dimer, ng/mL	770.5 (558.0‒1497.0)	840.0 (526.0‒1424.0)

^1^Values are mean ± SD, median (IQR), or n (%). Continuous variables were analyzed by independent *t*-test. Percentages were analyzed by Chi-Square or Fisher's exact test.

COVID-19, Coronavirus Disease 2019; BMI, Body Mass Index; ESR, Erythrocyte Sedimentation Rate.

a Pardo is the exact term used in Brazilian Portuguese, meaning “mixed ethnicity”, according to the Brazilian Institute of Geography and Statistics.

b BMI data was missing for 7.7 % of patients (*n* = 7 in the vitamin D_3_ group and *n* = 8 in the placebo group).

c Anticoagulant, antibiotic, glucocorticoid, antihypertensive, proton pump inhibitor, antiemetic, hypoglycemic, hypolipidemic, thyroid and antiviral data were missing for 0.5 % of patients (*n* = 1 in the vitamin D_3_ group).

d Analgesic data was missing for 1.0 % of patients (*n* = 1 in the vitamin D_3_ group and *n* = 1 in the placebo group).

e Computed tomography findings data were missing for 14.4 % of patients (*n* = 12 in the vitamin D_3_ group and *n* = 16 in the placebo group).

f Reference values: Platelet (150‒400/10^3^/µL); Mean platelet volume (9.4‒12.4 Fl); ESR (≤ 15 mm/h); C-reactive protein (< 5 mg/L); D-dimer (< 500 ng/mL).

In the vitamin D group, 84 patients (87 %) exhibited moderate disease, whereas 13 patients (13 %) had severe disease. In the placebo group, 86 patients (89 %) had moderate disease, while 11 patients (11 %) had severe disease.

As expected, mean (SD) 25-hydroxyvitamin-D was significantly increased from baseline to discharge after a single oral dose of 200,000 UI of vitamin D3 [from 21.1 (9.9) ng/mL to 44.5 (15.0) ng/mL] compared to placebo [from 20.0 (8.2) ng/mL to 19.5 (10.6) ng/mL] [significant group by time interaction (p < 0.001)]. No significant difference between vitamin D3 [3 (3.1 %)] and placebo [4 (4.1 %)] groups was observed in the frequency of thrombotic events (*p* = 0.700).

One hundred nine (66.5 %) patients tested positive for at least one type of aPL antibody. There was a significant group by time interaction (*p* = 0.046) for the frequency of aCL (IgG), with values increasing from baseline to discharge in the placebo group [from 13 (13.4 %) to 25 (25.8 %), *p* = 0.004], while frequency of aCL (IgG) in vitamin D_3_ group remained similar [from 25 (25.8 %) to 29 (29.9 %), *p* = 1.00], after Bonferroni's adjustment. However, the frequency of aCL (IgG) did not change between the groups on discharge. No significant differences between vitamin D_3_ and placebo groups were found for titers and frequency of aCL (IgM and IgA) and aβ2-GP (IgG, IgM and IgA) antibodies (Table 2).

**Table 2:** Effect of vitamin D_3_ on autoantibodies in patients with moderate to severe COVID-19.

Outcomes	Vitamin D_3_ group (*n* = 97)		Placebo group (*n* = 97)		p
	Baseline	Discharge	Baseline	Discharge	
aCL IgG, n (%)^a^	25 (25.8)	29 (29.9)	13 (13.4)^b^	25 (25.8)	0.046
aCL IgG, U/mL^a^	22.7 (11.4‒41.5)	26.0 (16.1‒48.6)	17.5 (11.5‒28.0)	25.6 (13.7‒41.4)	0.160
aCL IgM, n (%)	14 (14.4)	14 (14.4)	14 (14.4)	20 (20.6)	0.139
aCL IgM, U/mL^a^	17.3 (11.2‒30.0)	22.0 (14.6‒34.2)	16.4 (10.3‒29.3)	18.0 (13.3‒32.9)	0.550
aCL IgA, n (%)	6 (6.2)	7 (7.2)	9 (9.3)	8 (8.2)	0.559
aCL IgA, U/mL	14.7 (10.2‒22.8)	14.8 (11.1‒20.9)	17.0 (11.9‒24.8)	17.7 (11.2‒24.4)	0.097
aβ2-GP IgG, n (%)^a^	11 (11.3)	19 (19.6)	19 (19.6)	21 (21.6)	0.145
aβ2-GP IgG, U/mL	4.6 (2.8‒7.3)	4.7 (3.1‒8.8)	5.0 (2.9‒8.4)	5.6 (3.8‒9.8)	0.493
aβ2-GP IgM, n (%)	31 (32.0)	32 (33.0)	28 (28.9)	35 (36.1)	0.300
aβ2-GP IgM, U/mL^a^	7.1 (4.1‒10‒9)	7.7 (4.5‒11.8)	6.3 (3.8‒10.8)	8.1 (4.6‒12.5)	0.152
aβ2-GP IgA, n (%)	41 (42.3)	38 (39.2)	45 (46.4)	46 (47.4)	0.551
aβ2-GP IgA, U/mL	9.8 (6.9‒15.9)	10.3 (6.7‒15.8)	11.5 (7.3‒17.4)	11.3 (6.6‒18.2)	0.772

Data expressed as n (% within group) and median (IQR). Data were analyzed by Generalized Estimating Equations (GEE). aCL, Anticardiolipin; aβ2-GP, Anti-β2-Glycoprotein-I.

No significant differences were observed between groups at baseline; p-value derives from unadjusted group by time interaction from F-test.

a p < 0.05 for main effect of time.

b Significantly different (p < 0.05) from discharge in both groups.

## Discussion

To the best of our knowledge, this is the first double-blind, placebo-controlled, randomized clinical trial investigating the influence of 200,000 IU of vitamin D_3_ on aPL antibodies among hospitalized patients with moderate to severe COVID-19. Overall, no significant differences between vitamin D_3_ and placebo groups were found for any autoantibodies upon hospital discharge.

Antiphospholipid antibodies, such as aCL, aβ2-GP, and lupus anticoagulant, belong to a heterogeneous group of antibodies associated with Antiphospholipid Antibodies Syndrome (APS), a thrombosis-related systemic autoimmune disease affecting arteries, veins, and small blood vessels.^25^ It has been shown the presence of these antibodies in some viral infections,^26^, ^27^, ^28^, ^29^ among which COVID-19^6^ stands out, with a prevalence of aPL antibodies ranging from 3.7 % to 88.0 % in previous studies.^30^, ^31^, ^32^, ^33^ Similarly, herein the authors found that 66.5 % (129/194) of the patients showed at least one type of aPL antibody.

The immunomodulatory role of vitamin D appears to involve antigen-presenting cells, such as dendritic cells and macrophages, expressing a nuclear Vitamin D Receptor (VDR), a member of the nuclear receptor superfamily of transcriptional regulators involved in 1-alfa,25-dihidroxicolecalciferol signaling.^34^ However, the mechanisms underlying the inhibitory effects of vitamin D on antibody production are not fully understood. In part, this event could be explained by the regulatory effect of vitamin D on B-cells related to immune tolerance. Vitamin D sufficiency would act in the proliferation and apoptosis of activated B-cells, which are closely related to antibody production,^35^, ^36^, ^37^ while vitamin D deficiency would play the opposite effect, increasing the production of autoantibodies through the activation of B-cells.^37^^,^^38^

Riancho-Zarrabeitia et al.^39^ demonstrated an association between circulating vitamin D insufficiency (between 10 and 30 ng/mL) and the presence of lupus anticoagulant, an aPL antibody. When evaluating only patients with aPL antibodies syndrome, they showed an association between vitamin D insufficiency and higher frequency of anti-β2-glycoprotein-I, but not aCL.^39^

Another study assessed the effect of 50,000 IU of vitamin D in patients with Hashimoto's thyroiditis, a condition also characterized by autoimmune inflammation. Participants received the supplement weekly for three months and had a significant reduction in serum thyroid autoantibodies levels compared to baseline.^40^ Accordingly, an *in vitro* study^41^ showed that the 1,25-dihydroxyvitamin-D modestly reduced autoantibody production in peripheral blood mononuclear cells of disease-active systemic lupus erythematosus.

In patients with COVID-19, the presence of aPL antibodies has been suggested as one of the physiopathologic mechanisms for the cause of hypercoagulation and thrombotic events.^6^^,^^42^ In addition to promoting platelet aggregation and activation, aPL antibodies promote the upregulation of pro-inflammatory cytokines, cell adhesion molecules, and endothelial nitric oxide synthase that induce a pro-inflammatory and pro-coagulant endothelial phenotype.^43^

Notwithstanding, controversial results exist. In a prospective single-center observational study, patients with positive aPL antibodies did not have an increased risk of thrombosis risk during intensive care unit hospitalization.^32^ Additionally, Borghi et al.^44^ did not observe a significant correlation between the presence of aβ2-GP IgG and thrombosis, suggesting that aPL antibodies in COVID-19 may be different from those detectable in APS.^44^ Accordingly, the patients with aPL antibodies did not experience a high number of thrombotic events during hospitalization (4/133; *p* = 0.680), with no differences noted between the groups (*p* = 0.700). It is important to note that the presence of aCL in the present study was not significantly different between groups at discharge.

These conflicting results might be due to the fact that most of aPL antibodies associated with the virus are thought to be transient in patients with critical illness,^45^^,^^46^ so it is unclear whether either they represent a simple epiphenomenon or are actually involved in COVID-19-associated coagulopathy.^47^ In a study in which aPL testing was repeated 1 month after COVID-19 aPL-positive patients were admitted to the intensive care, aPL antibodies were primarily a transient phenomenon that occurred during the acute phase.^48^ Another confounded factor is that most patients were under anticoagulant medication, a drug that is related to the prevention of thrombotic events.

This study has some strengths, such as the comprehensive assessment of antibodies related to autoimmune diseases, the randomized, controlled, double-blinded design, and the enrollment of hospitalized patients with moderate to severe COVID-19. Also, these findings increase the knowledge on the role of vitamin D_3_ on aPL antibodies and better inform health professionals on more efficient healthcare delivery and resource management.

However, there are several limitations. First, the relatively small sample size could increase the chances of type 2 error. Second, the heterogeneity in pre-existing diseases and treatments of the patients, although no significant differences were observed between groups at baseline. Third, the results may have been influenced by the high rate of patients using anticoagulants (∼90 %). Finally, the authors were unable to measure other aPL antibodies, such as lupus anticoagulant antibodies.

In conclusion, these findings do not support the use of a single oral dose of 200,000 IU of vitamin D_3_ to modulate autoantibodies in hospitalized patients with moderate to severe COVID-19.

## Data availability

Deidentified participant data of this study must be requested from the corresponding author upon publication (sent to gualano@usp.br). The codebook of this study will be made available upon request by qualified clinical researchers for specified purposes dependent on the nature of the request and the intentional use of the data, with investigator support. The request must include a statistician. The lead author (BG) affirms that the manuscript is an honest, accurate, and transparent account of the study being reported; that no important aspects of the study have been omitted; and that any discrepancies from the study as originally planned (and, if relevant, registered) have been explained.

## Authors’ contributions

BG and RMRP: Had full access to all the data in the study and takes responsibility for the integrity of the data and the accuracy of the data analysis; LPS, LCVS and RMRP: Conceived and designed the study; LPS, LVBS, VFC and RMRP: Drafted the manuscript; LPS, ALF, BG, and RMRP: Performed statistical analysis; BG and RMRP: Provided supervision; LPS, MBGV, RMO, CPF and VFC: Provided administrative, technical, or material support; and all authors performed data acquisition, analysis, interpretation and critically revised the manuscript for important intellectual content. All authors read and approve the manuscript as submitted.

## Declaration of competing interest

The authors declare no conflicts of interest.
